# Hereditary angioedema and post traumatic stress disorder: a reciprocal relationship?

**DOI:** 10.1186/s13023-026-04403-5

**Published:** 2026-06-11

**Authors:** Sandra C. Christiansen, Dewleen G. Baker, Bruce L. Zuraw

**Affiliations:** 1 Division of Allergy and Immunology, Department of Medicine, University of California, 9500 Gilman Drive, MC#0732, La Jolla, San Diego, CA 92093 USA; 2 Center of Excellence for Stress and Mental Health, VASDHS, San Diego, CA USA; 3 Department of Psychiatry, UC San Diego, La Jolla, San Diego, CA USA; 4 Medicine Service, San Diego VA Healthcare System, San Diego, CA USA

**Keywords:** Post traumatic stress disorder, HAE due to C1 inhibitor deficiency, Depression, Stress, Quality of life, Beck depression inventory-II, PTSD checklist-civilian version, Childhood trauma questionnaire

## Abstract

**Background:**

Hereditary angioedema **(**HAE) patients endure repeated unpredictable trauma associated with attacks of disfiguring swelling, severe abdominal pain, risk of asphyxiation or experiences with loss of a loved one with HAE. Our study investigated the prevalence of presumptive post-traumatic stress disorder (PTSD) for HAE due to C1 inhibitor deficiency (HAE-C1INH).

**Results:**

Participants *≥* 18 with HAE-C1INH were recruited from the US HAE Association: Cohort 1, self-referred; Cohort 2, randomized from the HAEA membership. Participants completed 8 on-line questionnaires: (1) HAE survey; (2) SF-36; (3) Childhood Trauma Questionnaire (CTQ); (4) Beck Depression Inventory-II (BDI-II); (5) Life Events Checklist form for HAE; (6) PTSD checklist-civilian version (PCL-C); (7) Peritraumatic Dissociative Experiences Questionnaire (PDEQ) and (8) Peritraumatic Distress Inventory (PDI). The prevalence of presumptive PTSD based on the PLC-C outcomes was between 20 and 25% in HAE-C1INH participants (*n* = 77). Significant score correlations were found between: PLC-C and BDI-II (*p* < 0.0001, R^2^ = 0.23), total CTQ (*p* = 0.001, R^2^ = 0.13), PDEQ (*p* < 0.0001, R^2^ = 0.23) and PDI (*p* < 0.0001, R^2^ = 0.39). SF-36 total mental health scores were significantly associated with presumptive PTSD at all PLC-C cut-off scores as were all mental health subdomains, early age of HAE symptom onset and requiring emergency department visits for HAE attacks.

**Conclusion:**

Our study identified a high prevalence of presumptive PTSD among HAE-C1INH participants. Diagnosis was correlated with high rates of depression and reduced SF-36 mental domain performance. The interrelationship between PTSD and HAE may represent reciprocal risk factors enhancing disease severity.

## Introduction

Hereditary angioedema (HAE) due to C1 inhibitor deficiency (HAE-C1INH) is an ultra-rare autosomal dominant disease clinically characterized by attacks of swelling involving the subcutaneous tissue and mucous membranes. HAE attacks are unpredictable, often painful, disfiguring, and associated with an ever-present risk of asphyxiation and death [1, 2]. HAE can lead to psychological stress, reduced quality of life, and impairments in social, academic, and professional functioning [3–10].

An episode of extreme stress can be a traumatic event for some individuals. The defining characteristic of a traumatic event is its capacity to provoke fear, helplessness or horror in response to the threat of injury or death [11]. Individuals exposed to trauma are at increased risk of depression and anxiety along with mental health disorders such as panic disorder and substance abuse, compared with non-exposed individuals. Importantly a subgroup will also develop post traumatic stress disorder (PTSD). Factors that may predispose to PTSD can include life threatening events, uncontrollability, and unpredictability [12, 13]. These are unfortunately common features in the lives of individuals affected by HAE-C1INH.

In a large cohort study, 42.5% of HAE patients reported clinically significant depressive symptomatology that was linked to attack severity [3]. Patients also described consistently poorer health related quality of life (HrQOL) compared to healthy counterparts as well as a significant adverse impact on work and school. A 2004 survey found that HAE patients lived with the presence of constant fear of sudden closure of the airway, intolerable pain and transmission of HAE to children [14]. Despite substantial advances in therapy, a 2015 study documented that HAE patients experience persisting negative psychological effects, disruption in daily life, fear of suffocation and of having a child with HAE [15].

Individuals with HAE report a variety of factors that provoke attacks, with stress being among the most frequently cited. Mental stress was recorded as a triggering factor by 60% of HAE participants as well as the most frequently identified precipitant for swelling accounting for 21% of 3,176 attacks [16]. Stress has been linked to enhanced morbidity from a variety of diseases such as diabetes, cardiovascular disease, depression, obesity and asthma [17–21].

HAE typically presents in childhood, often with a significant delay in obtaining an appropriate diagnosis and access to effective therapy [1, 2, 22, 23]. Early experiences are believed to be a factor setting the level of responsiveness of the HPA axis and autonomic nervous system which are integral to the expression of PTSD [24]. As early age of occurrence as well as the cumulative burden or number of trauma events are linked to PTSD vulnerability these influences are expected to amplify the likelihood of PTSD in HAE patients [25].

We hypothesized that HAE-C1INH patients are at an enhanced risk for the development of PTSD. In this report we employed a validated screening instrument, the PTSD checklist-civilian version (PCL-C), to estimate the prevalence of PTSD in HAE patients. We further explored the relationship between PTSD, HAE disease severity, quality of life, childhood trauma and depression.

## Methods

### Participants

Participants with HAE-C1INH were recruited in collaboration with the United States Hereditary Angioedema Association (US HAEA). Given the rarity of HAE the best way to reach a representative population was through the patient advocacy organization (the US HAEA). The members are often well informed about their disease and as a group interested in studies which will increase our knowledge and thereby understanding of HAE leading to improved quality of life (QOL). Eligibility requirements were age *≥* 18 years of age, email account and access to the internet. There were no exclusion criteria. The study was conducted between 2013 and 2017 with IRB approval granted by The University of California San Diego.

Two separate cohorts were enrolled:

1. Self-referred cohort who responded to the announcement of the study at the 2013 US HAEA patient summit or to fliers posted on the US HAEA Facebook site and agreed to be contacted.
2. Randomized cohort selected from a randomized list of US HAEA members with a self-identified diagnosis of HAE-C1INH. Selected members received an email from the US HAEA inquiring whether they would consider participating. 91.8% of respondents agreed to participate.

Individuals from both groups who agreed to be contacted were sent detailed information about the study and called by one of the investigators (SCC) to verify the diagnosis, review the consent, and answer any questions. After receipt of the signed consent participants were emailed a secure link to begin participation.

### Questionnaires

Participants completed 8 questionnaires within 4 weeks in the following sequence: 1) *HAE survey* [26]; 2) *SF-36 QOL* [27, 28]; 3) *Childhood Trauma Questionnaire (CTQ)* [29]; 4) *Beck Depression Inventory-II (BDI-II)* [30, 31]; 5) *Life Events Checklist form* [32] *for HAE (LEC-HAE)*; *6) PTSD checklist-civilian version (PCL-C)* [33–47]; *7*) *Peritraumatic Dissociative Experiences Questionnaire (PDEQ)* [48, 49]; and *8) Peritraumatic Distress Inventory (PDI)* [50, 51]. Questionnaire descriptions, modifications/adaptations, scoring criteria and interpretation are detailed below.

#### Questionnaires

1. *HAE survey*: The survey was adapted from published consensus criteria to capture the participant’s HAE history and disease severity/morbidity (included in the on line repository) [26].
2. *SF-36 QOL*: The SF-36 is a validated instrument for evaluating QOL. It incorporates physical and emotional domains with eight scaled scores, which are the weighted sums of the questions in their Sects [27, 28].
3. *Childhood Trauma Questionnaire (CTQ)*: This is a validated 28 item instrument which generates an overall childhood trauma score created by summing five subscales (physical, emotional and sexual abuse, physical and emotional neglect) with possible scores ranging from 25 to 125 [29].
4. *Beck Depression Inventory-II (BDI-II)*: The BDI-II is a 21-item assessment utilized to capture the level of depression [30, 31]. Each item is representative of a particular symptom of depression and corresponds to diagnostic criteria listed in the DSM-IV. BDI-II scores are categorized as minimal depression (0–13), mild depression (14–19), moderate depression (20–28), or severe depression (29–63).
5. *Life Events Checklist form for HAE (LEC-HAE)*: The LEC-HAE (included in the on line repository) was adapted from the LEC (www.ptsd.gov), which has been previously evaluated in the predicted directions, with measures of psychological distress and strongly associated with PTSD [32]. The LEC-HAE facilitates identification of index traumas specific for HAE that could be used for assessment of PTSD symptoms with the PCL-C instrument (see below).
6. PTSD checklist-civilian version (PCL-C): The 17-item version of the PCL-C (DSM-IV) was linked to the participants’ most traumatic experience determined from the LEC-HAE. Scoring was done utilizing two standardized approaches to capture the symptom pattern and severity threshold for PTSD. The PCL-C was assessed by total severity score (range 17–85) [33]. Different PCL-C cut off scores have been used to make a PTSD diagnosis in a civilian primary care setting, varying from 26 to 50, with the appropriate cut-off dependent on the underlying prevalence of PTSD within the population being studied [33–42]. The PCL-C cut off score used to make a diagnosis of PTSD in a civilian primary care setting impacts the sensitivity and specificity of the tool. A value of 30 has a high sensitivity but sacrifices specificity. Conversely, use of higher cut-off scores, increases specificity with a corresponding loss of sensitivity [33]. A revised PCL-5 instrument using the DSM-V criteria has been developed. While not interconvertible, the PCL-C and PCL-5 have been shown to yield comparable results [47]. We utilized DSM IV criteria for the diagnosis of PTSD rather than the revised criteria in DSM V [11]. The revised DSM V criteria have engendered considerable debate in the field of psychiatry [52]. In contrast, the World Health Organization’s International Classification of Diseases, 11th Revision (ICD-11) closely mirrors the DSM-IV criteria for PTSD. The implications of the DSM-V PTSD criteria have yet to be fully realized. There is an appreciation however that the revision should not be utilized to invalidate the diagnosis of PTSD by DSM-IV criteria [52].
  In addition to the total severity score, the PCL-C can be used to screen for PTSD using a score of 3 or more in: (1) one item from questions concerning persistent reexperiencing of the trauma (cluster B, questions 1–5); (2) three items from questions concerning persistent avoidance of stimuli (cluster C, questions 6–12); and 3); two items from questions regarding persistent increased arousal (cluster D, questions 13–17). This is referred to as the B + C+D criteria.
7. *Dissociative Experiences questionnaire*: This questionnaire combines the standardized Peritraumatic Dissociative Experiences Questionnaire (PDEQ) and the Peritraumatic Distress Inventory (PDI) into a single questionnaire that is linked to the most traumatic experience form the LEC-HAE as is the case for the PCL.

### Online survey

Survey instruments were hosted on the UCSD REDCap iDASH site. Participants were emailed a unique password and a link to complete the first questionnaire. Sequential invitations were sent after completion with links and instructions for the next questionnaire. A small incentive ($5 gift card) was offered for each completed questionnaire.

### Data analysis

The primary endpoint was the estimated prevalence of PTSD. Secondary aims were to examine the relationship between PLC scores and other measures, including trauma exposure, HAE-C1INH morbidity, childhood trauma, QOL, criteria for depression, and measures of dissociation (PDEQ and PDI). Participants who did not complete all questionnaires were excluded from the analysis.

Nominal data was analyzed using contingency tables with Fisher’s exact test. One way analysis of continuous data was analyzed non-parametrically with the Wilcoxan test. Bivariate data was analyzed by linear regression using analysis of variance. Multiple linear regression was used to assess the relative strength of the different model factors. Residuals were examined including studentized residuals, residual normal quantile plot, and Durbin-Watson test to exclude heteroscedasticity, non-linearity, and autocorrelation. Statistical tests were performed using JMP 18.2.1 (SAS, Cary NC).

## Results

### Participant demographics

Eighty-two HAE-C1INH participants were enrolled. Five did not complete all questionnaires and were eliminated. Of the 77 participants analyzed, 29 were self-referred and 48 were randomized. Table 1 summarizes their demographic and clinical information. There were no significant differences between the two cohorts, although there was a near significant difference in the age of first attack with the self-referred group reporting an earlier age.

**Table 1:** Demographics of HAE-C1INH participants

	Combined	Self-Referred	Random	Self-Referred vs. Random
Number participants (#)	77	29	48	*p*-value
Enrollment Age^$^	51 (39.5,57)	50.5 (45,57.8)	51 (37.3,57)	0.86
Female/Male	63/14	24/5	39/9	1.00
Age^$^ at symptom onset	11 (7,15)	10 (5,13)	12 (8.3,16)	0.057
# IV pd C1INH* (Berinert)	23	12	11	n/a
# pd IV C1INH* (Cinryze)	42	16	26	n/a
# Ecallantide (Kalbitor)	4	1	3	n/a
# Icatibant (Firazyr)	49	17	32	n/a
# rhC1INh** (Ruconest)	3	0	3	n/a
# AA***	13	3	10	n/a
# symptomatic days prior 12 months	68 (33,185)	59 (33,182)	69 (32,198)	0.69
Recall, symptomatic days worst 12-month period	129 (57,310)	156 (68,468)	104 (56,235)	0.24

# Number; ^$^Years; *Intravenous plasma derived C1 Inhibitor; **Recombinant C1INH; ***Attenuated androgens

### Prevalence of presumptive PTSD in HAE participants

In the absence of Clinician Administered PTSD Scale (CAPS) interviews, the PLC-C (reflecting DSM-IV symptoms of PTSD) can be used to screen populations for the prevalence of PTSD. We analyzed the PLC-C data utilizing both the total score and the B + C+D method.

The appropriate PLC-C cut-off score for a diagnosis of PTSD (typically 30–50) has been shown to vary based on the prevalence of PTSD within that population [33]. Since the underlying frequency of PTSD within the HAE population is unknown, we assessed our cohort using graded levels of PLC-C cut-off stringencies (Table 2). The percentage of participants with presumptive PTSD varied from 48.6% using a cut-off score of 30 (high sensitivity, low specificity) down to 18.1% using a cut-off score of 50 (low sensitivity, high specificity).

**Table 2:** Prevalence of presumptive PTSD in HAE-C1INH

Criteria	Combined Participants	Self-Referred Cohort	Random Cohort	Self-Referred vs. Random Cohorts
	*n* = 72	*n* = 24	*n* = 48	*p*-value*
PCL-C ≥ 30	35 (48.6%)	15 (62.5%)	20 (41.7%)	0.134
PCL-C ≥ 35	27 (37.5%)	13 (54.2%)	14 (29.2%)	0.069
PCL-C ≥ 40	19 (26.4%)	10 (41.7%)	9 (18.8%)	0.049
PCL-C ≥ 45	15 (20.8%)	7 (29.2%)	8 (16.7%)	0.234
PCL-C ≥ 50	13 (18.1%)	6 (25.0%)	7 (14.6%)	0.336
B + C+D	15 (20.8%)	7 (29.2%)	8 (16.7%)	0.234

* Contingency table, 2 × 2 Chi-square Fisher’s exact test

**Fig. 1:**
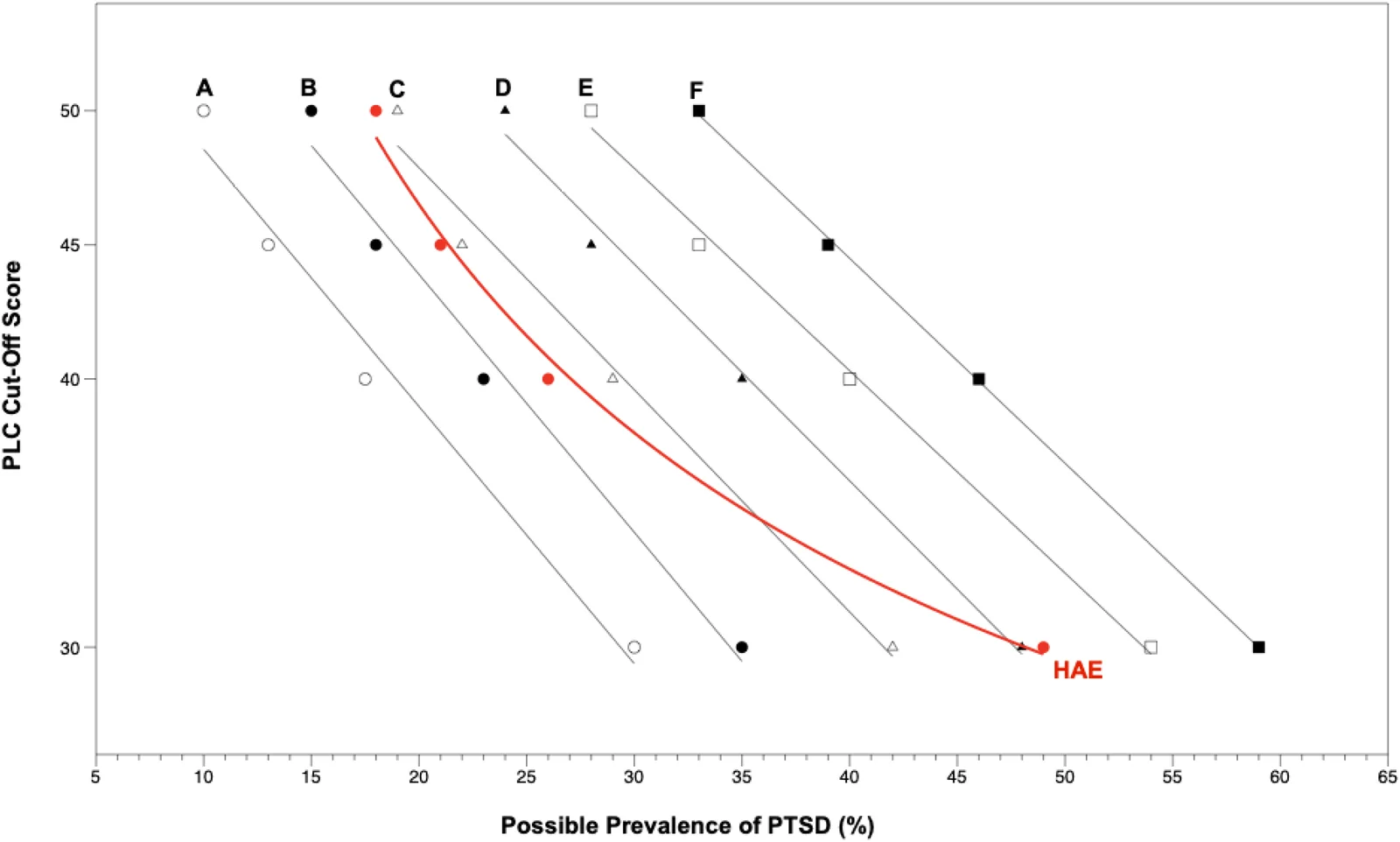
Impact of underlying population PTSD prevalence on the accuracy of PTSD diagnosis using the PLC score. Data from Terhakopian et al. [33] was plotted showing the linear relationships between the PLC cut-off scores (ordinate) and the population PTSD prevalence (abscissa). Six populations were modeled with underlying PTSD prevalences of: A, 5%; B, 15%; C, 25%; D, 35%; E, 45%; and F, 55%. Data from the HAE-C1INH cohort (in red) was overlaid on the data from Terhakopian et al. and most closely resembles population C with an underlying PTSD prevalence of 25%

To refine the estimate of presumptive PTSD prevalence in HAE-C1INH, we utilized data from Terhakopian et al. in which the estimated sensitivity and specificity of various PLC-C cut-off scores were assessed across 6 different populations with underlying PTSD prevalences ranging from 5 to 55% [33]. By plotting PLC-C cut-off score against the underlying population prevalence of PTSD (Fig. 1), we found that the HAE population most closely resembled the Terhakopian population with a prevalence of 25%.

We also calculated the prevalence of presumptive PTSD using the B + C+D criteria, suggested to be the equivalent of a PLC-C cut-off score of 50 or higher [43–46]. Based on the B + C+D criteria, we measured a presumptive prevalence of PTSD in our HAE cohort of 20.8%.

We next compared the presumptive prevalence of PTSD between the self-referred and random groups. The self-referred group displayed a higher percentage of presumptive PTSD at all stringency levels; however, the differences did not meet statistical criteria with the exception of a minimally significant difference at the 40 cut-off (Table 2). Therefore, we used the entire cohort for the remaining analysis.

### Trauma exposure

**Fig. 2:**
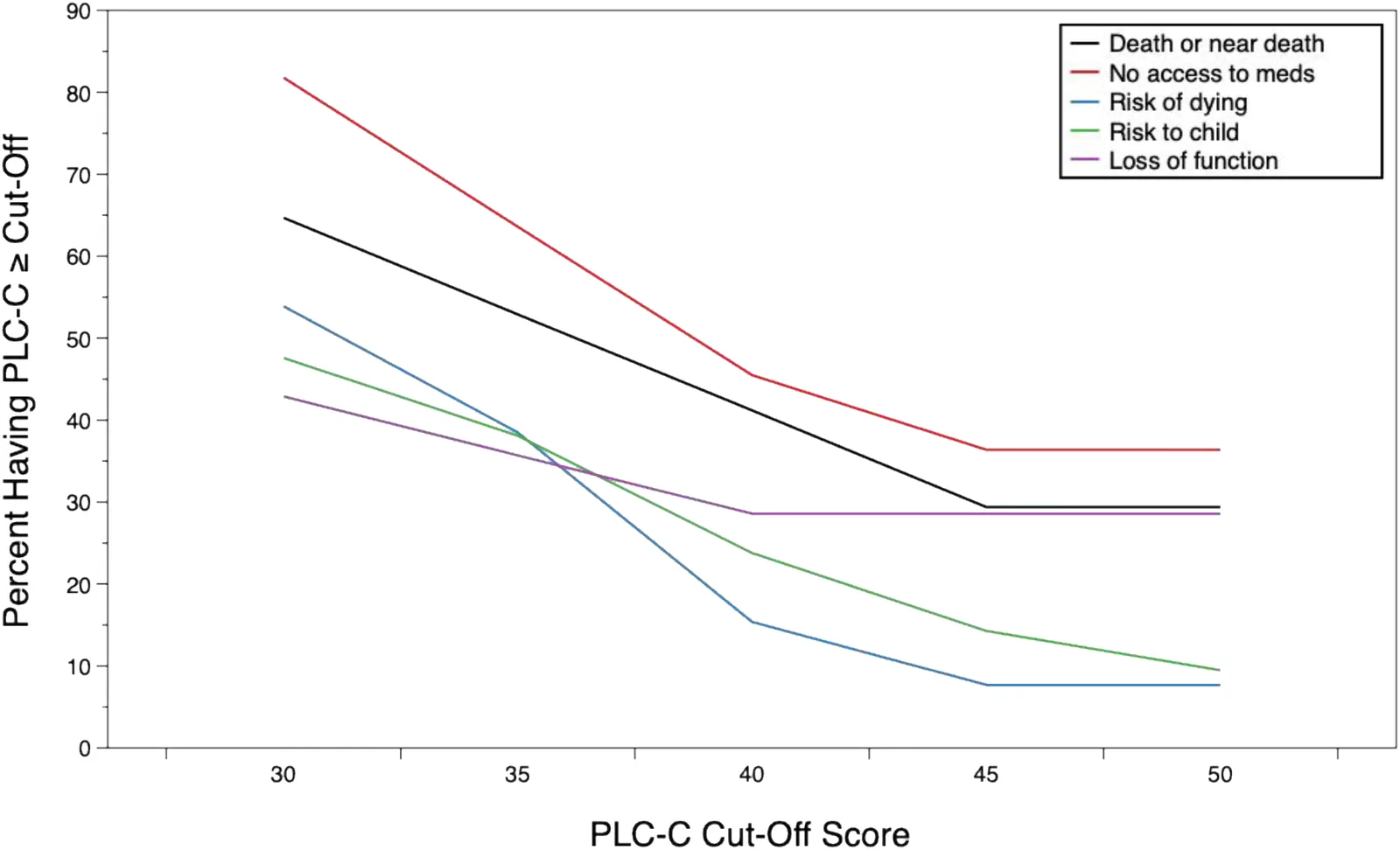
Percent of HAE-C1INH participants reporting specific traumas who met the PLC-C criteria for presumptive PTSD. The five most commonly reported HAE-related traumas are shown. For each of these traumas, the percent of HAE-C1INH participants (ordinate) reporting that trauma who met or exceeded the PLC-C cut-off score from 30 to 50 (abscissa) are plotted

Participants identified their 3 most severe traumas on the LEC-HAE questionnaire. Table 3 displays the HAE events identified by the participants as their 3 most significant as well as their single worst trauma. The most commonly identified traumas were risk of a child developing HAE; death or near-death from a HAE attack; impaired functioning during attacks; the risk of dying from an attack; and the lack of access to effective treatment. Figure 2 shows the percent of participants identifying each of these five traumas who had a PLC-C score at or above the cut-off using scores from 30 to 55.

**Table 3:** Exposure to HAE related traumatic events

	% Reporting	% Reporting when < 18 y/o	Median frequency or severity	Three worst, *n* (%)	Worst, *n* (%)
Near-death experience or death of relative from HAE	71.4%	47.1%	Couple of times	17 (22.1%)	10 (13.0%)
Fear of suffocation during attack	85.7%	42.9%	Number of times	9 (11.7%)	6 (7.8%)
Difficulty swallowing from laryngeal attack	82.9%	82.4%	Number of times	4 (5.2%)	0
Distressing pain from attack	100%	85.7%	Too many times to count	8 (10.4%)	2 (2.6%)
Emergency intubation for attack	34.3%	42.9%	Couple of times	4 (5.2%)	2 (2.6%)
Unnecessary surgery due to attack	40%	28.6%	Once	1 (1.3%)	0
Emergency hospitalization for attack	94.3%	63.3%	Many times,	2 (2.6%)	1 (1.3%)
ED visit for attack	97.1%	60.6%	Too many times to count	3 (3.9%)	1 (1.3%)
Fainting or almost fainting from attack	74.3%	69.2%	Many times,	2 (2.6%)	0
Embarrassment due to disfiguration from attack	88.6%	73.3%	Too many times to count	5 (6.5%)	0
Difficulty urinating or defecating from attack	71.4%	60%	Too many times to count	0	0
Distressing vomiting from attack	97.1%	79.4%	Too many times to count	2 (2.6%)	0
Accused of drug-seeking behavior during attack	42.9%	7.1%	Number of times	3 (3.9%)	0
Misdiagnosed with psychiatric disorder during an attack	60%	57.9%	Many times,	2 (2.6%)	0
Physician treating attack did not understand the disease	94.3%	61.3%	Too many times to count	5 (6.5%)	0
Received inappropriate treatment for attack	91.4%	64.5%	Many times,	4 (5.2%)	1 (1.3%)
Inability to access necessary medication	65.7%	65.2%	Many times,	11 (14.3%)	6 (7.8%)
Concern that would die from HAE	80%	46.4%	Extreme severity	13 (16.9%)	4 (5.2%)
Concern about having a child due to HAE	65.7%	18.2%	Severe concern	21 (27.3%)	6 (7.8%)
Feeling isolated due to HAE	85.7%	58.6%	Moderately severe concern	2 (2.6%)	0
Concerned about ability to function in life	88.6%	38.7%	Severe concern	14 18.2%)	4 (5.2%)
Concern about side effects from HAE medicine	71.4%	28%	Moderate concern	1 (1.3%)	0

Forty-two participants identified at least one non-HAE trauma as one or more of their 3 most severe traumas. The most common non-HAE traumas were death of a close relative [16], other serious illnesses [14], interpersonal family strife [11], financial stress [8], physical or emotional abuse [8], and pregnancy complications [5].

### Relationship between PLC-C score and BDI II, CTQ, PDEQ, PDI

We examined the correlation between the PLC-C score and other measures of psychologic distress, including depression (BDI II), childhood trauma (CTQ), dissociative symptoms (PDEQ), and distress (PDI) in our HAE population measured by regression and ANOVA analysis. Significant correlations were found between the PLC-C scores and BDI scores (*p* < 0.0001, R^2^ = 0.23), total CTQ scores (*p* = 0.001, R^2^ = 0.13), PDEQ scores (*p* < 0.0001, R^2^ = 0.23) and PDI scores (*p* < 0.0001, R^2^ = 0.39).

The relationship between each of these measures of psychologic stress and presumptive PTSD diagnosis was further evaluated at the different PLC-C cut-offs (Table 4). Depression (BDI II ≥ 29) was significantly associated with presumptive PTSD diagnosis at all cut-off scores. Individual analysis of CTQ sub-scores revealed a significant presumptive PTSD correlation with emotional abuse at all PLC-C cut-offs. In contrast, Physical abuse, and emotional neglect were not significantly associated. Sexual abuse was correlated with presumptive PTSD at higher PLC-C stringencies (≥ 40), which was significant at cut-offs of 40, 50, and B + C+D. Physical neglect was also significantly associated with presumptive PTSD at PLC-C cut-offs of 40 and above. Trauma-related dissociation (PDEQ) and distress (PDI) have been closely linked with the development of PTSD. We found that both measures were significantly associated with presumptive PTSD at all PLC-C cut-offs.

**Table 4:** Correlation between measures of psychologic distress and presumptive PTSD

	*p*-value* at indicated PTSD criteria					
	≥ 30	≥ 35	≥ 40	≥ 45	≥ 50	B + C+D
Depression ^§^	**< 0.001**	**0.001**	**0.004**	**0.001**	**< 0.001**	**0.001**
Emotional abuse ^¶^	**0.018**	**0.003**	**0.009**	**0.001**	**< 0.001**	**0.008**
Physical abuse ^¶^	0.802	0.603	0.582	1.000	0.743	0.537
Sexual abuse ^¶^	0.619	0.316	**0.012**	0.121	**0.025**	**0.028**
Emotional neglect ^¶^	1.000	1.000	0.557	0.521	0.307	0.200
Physical neglect ^¶^	0.141	0.130	**0.028**	**0.013**	**0.010**	**0.013**
PDEQ ^#^	**< 0.001**	**< 0.001**	**< 0.001**	**< 0.001**	**< 0.001**	**< 0.001**
PDI °	**< 0.001**	**0.001**	**< 0.001**	**0.002**	**0.006**	**0.002**

* Chi-square exact test

^§^ Contingency tables of BDI II assessed depression (minimal/mild versus moderate/severe) and presumptive PTSD (negative versus positive) using the different cut-off criteria

^¶^ Contingency tables of CTQ subscales (maltreated versus not maltreated) and presumptive PTSD (negative versus positive) using the different cut-off criteria

^#^ Contingency tables of PDEQ (low dissociation versus high dissociation) and presumptive PTSD (negative versus positive) using the different cut-off criteria

^°^ Contingency tables of PDI (non-distressed versus distressed) and presumptive PTSD (negative versus positive) using the different cut-off criteria

### Relationship between PLC-C score and SF-36 QOL

The relationships between quality of life and prevalence of screening for PTSD was assessed using the SF-36 instrument (Table 5). The total mental health score was significantly associated with presumptive PTSD at all PLC-C cut-off scores. Further, all mental health subdomains were also significantly associated with presumptive PTSD at all cut-off scores. The total physical health score was significantly associated with presumptive PTSD at cut-off scores ≥ 45, and the physical health subscales were generally significantly associated with presumptive PTSD at PCL-C cut-off scores ≥ 35. Similar to results found in Persian Gulf War Veterans with PTSD [53, 54], HAE participants with both presumptive PTSD and depression had significantly lower vitality scores (*p* < 0.0001). No significant differences between men and women were found for PLC-C, BDI II, CTQ, PDEQ, or PDI scores.

**Table 5:** Relationship between SF-36 scales and PTSD in HAE

SF-36 scale	*P*-values* vs. PTSD Criteria							
	≥ 30		≥ 35		≥ 40	≥ 45	≥ 50	B + C+D
Total mental health	< 0.001*		**< 0.001**		**0.003**	**0.003**	**0.006**	**0.003**
vitality	**0.004**		**< 0.001**		0.060	**0.029**	**0.009**	**0.044**
social functioning	**< 0.001**		**< 0.001**		**0.008**	**0.004**	**< 0.001**	**< 0.001**
role emotional	**< 0.001**		**< 0.001**		**0.001**	**< 0.001**	**< 0.001**	**< 0.001**
mental health	**< 0.001**		**< 0.001**		**0.007**	**0.012**	**0.023**	**0.01**
Total physical health	0.444		0.159		0.089	**0.035**	**0.004**	**0.008**
physical functioning	0.098		**0.023**		**0.026**	**0.031**	**0.005**	**0.003**
role physical	0.065		**0.017**		**0.010**	**0.002**	**< 0.001**	**< 0.001**
bodily pain	0.134		**0.049**		0.095	**0.031**	**0.002**	**0.031**
general health	**0.013**		**0.011**		**0.031**	**0.010**	**0.007**	**0.005**

* two-way Wilcoxon test

### Relationship between HAE-C1INH morbidity and PTSD

Next, we examined how HAE morbidity impacted the likelihood of meeting PCL-C screening criteria for PTSD utilizing two parameters of HAE severity.

*Age of first angioedema attack and delay to diagnosis*. Earlier age of symptom onset has been repeatedly linked to more severe HAE [22]. Consistent with this, we found an inverse relationship between the age of first angioedema attack and the PLC-C score. The mean ± SEM PLC-C score for all participants was 30.61 ± 1.97. PLC-C scores were analyzed using the Wilcoxon Rank Sum test. The mean PLC-C scores stratified on the age of first angioedema attack were 27.62 ± 3.04 in participants ≥ 12 years versus 32.98 ± 2.57 in participants < 12 years (*p* = 0.047), 29.53 ± 2.22 in participants ≥ 6 years versus 35.07 ± 4.23 in participants < 6 years (*p* = 0.147), and 29.26 ± 2.06 in participants ≥ 3 years versus 44.14 ± 4.38 in participants < 3 years (*p* = 0.009). In a regression analysis, PLC-C score increased as the delay in diagnosis increased, but the effect was not statistically significant.

*Angioedema attacks requiring Emergency Department (ED) Treatment*. The mean ± SEM PLC-C score was compared between participants who reported needing Emergency Department treatment of angioedema attacks (*n* = 59) versus those who did not require ED treatment (*n* = 17). The PLC-C was significantly higher in those requiring ED treatment (33.29 ± 2.26) compared to those not requiring ED treatment (23.12 ± 3.31; *p* = 0.007, Wilcoxon rank sum). Participants requiring ED treatment also had significantly higher dissociation scores (PDEQ and PDI) scores. SF-36, CTQ, or BDI II scores did not significantly differ.

*Medications*. The medications used for on-demand and long-term prophylaxis in our participants is shown in Table 1. We found no impact of any of these medications on the PLC-C score.

### Multiple regression analyses

A multiple regression analysis was performed using model factors of PDI, PDEQ, BDI II, CTQ, SF-36 mental health score, SF-36 physical health score, age of first attack, delay in diagnosis, the interaction between age of first attacks and delay in diagnosis, the number of ED visits, disease severity in the last 12 months, and worst disease severity to predict the dependent PLC-C score (adjusted R^2^ = 0.61; *p* < 0.0001). The independent model factors with the largest and statistically significant effects were PDI score, delay to diagnosis, and number of ED visits. We also built a multivariate model containing each of the LEC-HAE factors and found no significant predictive effect on the dependent PLC-C score.

## Discussion

HAE patients suffer from repeated attacks of angioedema with attendant risk of significant morbidity and potential mortality. Symptoms typically begin during childhood and worsen with the onset of puberty following a variable course over a patient’s lifetime Because HAE is an autosomal dominant disease with high penetrance, individuals often bear witness to family members suffering and sadly dying from the disease throughout their lifetime. The diagnosis of HAE is often missed by physicians for protracted periods of time, subjecting patients to treatment with ineffective medicines, accusations of histrionic or drug-seeking behavior, thereby substantially increasing the morbidity and mortality of the disease [22, 23, 55]. The delay between onset of symptoms and correct diagnosis on average was ~ 22 years in a 1976 study [56] and remains over 8 years in the United States [22]. The characteristic features of HAE with childhood onset, unpredictable nature of attacks, lack of controllability and cumulative trauma burden formed the basis for our hypothesis that HAE-C1INH patients may be vulnerable to PTSD.

PTSD has been estimated in the National Comorbidity Survey Replication using DSM IV criteria to have a lifetime prevalence for adult Americans of 6.8% with a current past year prevalence of 3.5% [57, 58]. A 2008 survey of 1,938 veterans who served in Operation Iraqi Freedom (OIF) or Operation Enduring Freedom (OEF) (deployed in Afghanistan and Iraq) had a 13.5% overall incidence of PTSD based on PCL screening [59]. The definitive test to establish a diagnosis of PTSD requires a structured diagnostic interview such as the clinician administered PTSD Scale (CAPS). For many studies, a probable diagnosis of PTSD can be assessed using the validated self-administered PLC-C questionnaire [43]. In the absence of a CAPS interview the diagnostic category of post-traumatic stress (PTS) which is considered a subcategory of PTSD, can be established using the PLC score alone. The prevalence of PTS in the United States is estimated at 6% in the general population. For this report, we are using the designation of presumptive PTSD which is equivalent to PTS. A parallel relationship between increasing disability and symptoms of PTS has been demonstrated, even when the threshold criteria for PTS disorder have not been met [60–62].

Two different approaches have been developed to score the PLC-C: (1) summing all 17 items and selecting a cut-off score; or (2) meeting the DSM-IV criteria of moderate or higher distress in at least one reexperiencing symptom, three avoidance symptoms, and two hyperarousal symptoms (B + C+D). In this report we provide the first attempt to quantify the prevalence of presumptive PTSD in HAE-C1INH using the PCL-C.

Cut-off scores used to diagnose probable PTSD in civilian populations have ranged from 26 to 50 [41, 63], with lower cut-offs having greater sensitivity and higher cut-offs having greater specificity. We found that the prevalence of presumptive PTSD in our HAE-C1INH cohort varied from 48.6% using a PLC-C cut-off score of 30 to 18.1% using a PLC-C cut-off score of 50 (Table 2). Utilizing data from a previous study that employed both the PLC-C and structured diagnostic interviews, (CAPS), in populations with different PTSD prevalences [33], we found that the HAE-C1INH cohort closely modeled the population with a PTSD prevalence of 25%. Using the B + C+D scoring method, we found a presumptive PTSD prevalence of 20.8%, which equated to a PLC-C cut-off score of 45. Based upon the aggregated results of these methodologies, we conclude that the underlying prevalence of PTSD in HAE-C1INH is likely between 20 and 25%.

To determine whether the high prevalence of PTSD in the self-referred cohort could be influenced by selection bias, we analyzed the incidence of PTSD by PCL-C criteria in 48 individuals with HAE-C1INH randomly identified from the membership of the US HAEA. 41.7% met criteria for presumptive PTSD by ≥ 30 scoring and 16.7% by B + C+D criteria. No significant difference was detected between the self-referred and random cohorts by either PCL-C criterion, suggesting a very high presumptive prevalence of PTSD in the overall HAE population.

PTSD is defined as resulting from traumatic events that involve actual or threatened death, serious injury, or sexual violence. Initially studied in military populations, PTSD has increasingly been recognized in civilian populations exposed to other forms of non-kinetic trauma, including medical trauma, psychologic violence, interpersonal trauma, unexpected death of a loved one, developmental trauma, and identity-based trauma [64–71]. We inquired about the most significant HAE-related traumas experienced in our cohort. Not surprisingly, near-death experience of the participant or an unexpected death of a relative was frequently identified. The other most frequently identified HAE-related traumas were the risk of a child developing HAE, risk of dying from an HAE attack, impaired functioning due to HAE, and the lack of access to effective HAE treatment. Notably the HAE-related trauma most highly correlated with a presumptive PTSD diagnosis was the lack of access to effective treatment (Fig. 2).

We examined how other determinants of psychologic stress in our HAE-C1INH cohort correlated with PTSD. In previous studies, HAE has been associated with significant stress, anxiety, depression, and decreased quality of life [3–10]. Measures of dissociation and distress are closely related to PTSD and unsurprisingly were significantly correlated with PLC-scores and presumptive PTSD diagnosis. Quality of life SF-36 scores were associated with presumptive PTSD, most significantly in mental health domains. A significant correlation was noted between moderate to severe depression and PTSD. Participants with both depression and PTSD displayed a striking decrease in SF-36 vitality. We further detected a correlation between childhood emotional abuse or sexual abuse with presumptive PTSD. Taken together our results support a profound psychological burden for patients suffering from HAE. It is generally accepted that children exposed to trauma represent a high-risk group for the development of PTSD [72]. Given the typical childhood onset of symptoms coupled with the large majority of patients having a family member affected by HAE-C1INH, the possibility of experiencing major trauma at a young age is substantial possibly translating into an elevated prevalence of PTSD.

We analyzed the relationship between several measures of HAE-C1INH morbidity and the PLC-C score. The age of symptom onset revealed a significant inverse relationship with the PLC-C score. This mirrors a prior report demonstrating that HAE-C1INH patients who develop symptoms at an early age experience greater disease morbidity [22]. We also found a significant relationship between the need for HAE treatment in the emergency department and the PLC-C score.

Current treatment status was not significantly related to the PLC-C score in our cohort. The relatively recent availability of highly effective HAE treatments may, however, have limited our ability to detect an effect during the course of our investigation. Conversely, measures of ongoing stress were found to be associated with PTSD in keeping with the long-standing recognition of the adverse impact of stress on HAE-C1INH outcomes [2, 16, 73].

Highly effective long term prophylactic therapies (LTP) approved since the conclusion of our study (subcutaneous C1INH, lanadelumab, berotralstat, garadacimab and donidalorsen [74]) have demonstrated a positive impact on QOL [75, 76]. The treatment paradigm has shifted towards LTP incorporation thereby enabling individuals with HAE to lead an essentially “normal” life. Future HAE generations may hopefully avoid the deleterious psychological and emotional sequelae that individuals have suffered in the past.

Mechanistically, PTSD and HAE may reciprocally influence one another through the release of cytokines or mediators involved in both neuropsychological and immunological signaling. HAE-C1INH patients display evidence of constant generation of bradykinin which spikes during attacks [77]. Elevated levels of bradykinin cause chronic stimulation of the sympathetic nervous system which has been linked to enhanced risk of developing PTSD [78, 79]. Elevated levels of IL-1β and IL-6 reported in depression and PTSD [80] may contribute to heightened HAE severity. IL-1β upregulates expression of the inducible bradykinin B1 receptor, which may be involved in HAE-C1INH attacks [81]. IL-6 regulates the acute phase response and leads to upregulation of fibrinogen and heat shock protein 90 (HSP90). Fibrinogen potentiates bradykinin-dependent vasodilation, the genesis of angioedema attacks [82] HSP90 in turn enhances activation of the plasma kallikrein-kinin system thereby generating bradykinin, the mediator of swelling [83, 84]. IL-1β and IL-6 could thus orchestrate an increased frequency and severity of attacks in keeping with the observed relationship between HAE and stress. Alterations in autonomic nervous system modulation in HAE-C1INH patients, specifically increased sympathetic activation at rest and blunted response to orthostatic challenge, would also be consistent with the influence of IL-1β and IL-6, providing an intriguing although speculative link between stress and bradykinin production [85]. Indeed, a hallmark specific for PTSD mirrors a finding identified in HAE; autonomic nervous system imbalance, characterized by a hypoactive parasympathetic nervous system and hyperactive sympathetic nervous system [86–88].

Our study was the first to attempt to explore the intersection between development of the mental health disorder PTSD and HAE. A weakness of our investigation was the lack of diagnostic confirmation by administration of the Clinician Administered PTSD Scale, considered the gold standard for diagnosis [46]. We therefore qualified findings by “presumptive” as applied to our reported outcomes.

In conclusion, individuals with HAE appear to be at disproportionate risk for developing PTSD, depression and impairments in emotional/mental domains for quality of life. Further research to explore and confirm the relationship between HAE and PTSD is expected to provide meaningful insights into mechanisms of disease and advance the foundation for novel treatment approaches.

## Data Availability

The data supporting the findings of this study are available from the corresponding author upon reasonable request.

## Abbreviations

BDI II: Beck Depression Inventory-II
C1INH: C1 Inhibitor
CTQ: Childhood Trauma Questionnaire
HAE: Hereditary Angioedema
LEC-HAE: Life Events Checklist form for HAE
QOL: Quality of Life
HrQOL: Health Related Quality of Life
PCL-C: PTSD checklist-civilian version
PTSD: Post Traumatic Stress Disorder
